# The Off-Label Use of a Humeral Nail To Treat a Subtrochanteric Femur Fracture: A Case Report

**DOI:** 10.7759/cureus.77505

**Published:** 2025-01-15

**Authors:** Alison Buseck, Michael Koulopoulos, Matthew P Sullivan

**Affiliations:** 1 Orthopedic Surgery, State University of New York Upstate Medical University, Syracuse, USA

**Keywords:** congenital hip dysplasia, femoral neck osteotomy, femoral neck-shaft angle, im nail, subtrochanteric femur fracture

## Abstract

The use of humeral nails for femoral subtrochanteric fractures is rarely reported. We present a case in which a humeral nail was employed for a subtrochanteric femur fracture alongside contralateral prophylactic stabilization of the femoral neck and shaft.

A 29-year-old female patient with a history of bilateral varus-producing proximal femoral osteotomies, complicated by symptomatic hardware indicating bilateral removal, sustained a subtrochanteric fracture. Given her 98-degree neck-shaft angle, a humeral nail was selected for right femur fixation. She also underwent prophylactic stabilization of the contralateral proximal femur with a humeral nail.

There are limited options for addressing subtrochanteric femur fractures in patients with anatomy variations that preclude using standard femoral nails. This case demonstrates the successful off-label use of a humeral nail for subtrochanteric femur fracture when a traditional femoral nail is impossible.

## Introduction

Subtrochanteric femur fractures are effectively stabilized with intramedullary nails and, to a lesser degree, plate-and-screw constructs [1]. Alternatives to intramedullary nailing for femoral shaft fractures include plate osteosynthesis, which often requires greater soft tissue disruption, carries higher complication rates, and may be unsuitable for highly comminuted fractures [2]. Femoral cephalomedullary nails are designed based on a defined neck-shaft angle. Nail geometry is based on the normal anatomic range of the neck-shaft angle, typically around 125-130°. In patients with anatomy outside this range, safe placement of cephalomedullary nails may not be feasible [3]. Studies have explored the off-label use of proximal humerus locking plates for proximal femur fractures when traditional fixation methods are not an option [4]. These plates can be used when the neck-shaft angle approaches 90°, as they accommodate such anatomical deviations [4]. However, the mechanical limitations of locked plate and screw constructs are significant, with failure rates reported as high as 36% in proximal femur fixation [5]. In contrast, the humeral nail allows fixed-angle fixation for the femoral head and neck, similar to a proximal humeral plate, but with the added benefits of an intramedullary device that more closely aligns with the femoral mechanical axis than surface-based plate-and-screw constructs. This makes the humeral nail an appealing option for patients with markedly abnormal femoral neck-shaft angles in the setting of a subtrochanteric femur fracture, which would typically be treated with a cephalomedullary device. To the best of our knowledge, reports on humeral nails used in femoral subtrochanteric fractures are limited. Existing literature on humeral nail use for femoral fractures primarily describes cases in children and adolescents with femoral shaft fractures [6], a paraplegic patient with a subtrochanteric femoral fracture aimed at achieving positional stability and minimizing soft tissue damage [7], and femoral reconstruction in adolescents with osteogenesis imperfecta [8]. We present a case in which a humeral nail was used for a subtrochanteric femur fracture and contralateral prophylactic stabilization of the femoral neck and shaft in a patient with prior varus-producing proximal femoral osteotomies. 

The patient was informed that data from her case would be submitted for publication, and she provided consent.

## Case presentation

We describe the case of a 29-year-old female patient with a medical history significant for congenital hip dysplasia. Her orthopedic care began at age 19 when she underwent bilateral varus-producing proximal femoral osteotomies with 95° blade plates for fixation. This was complicated by painful hardware, necessitating bilateral removal. The patient later developed a left subtrochanteric stress fracture two months after hardware removal, followed by a right subtrochanteric fracture seven years later. At the time of the left stress fracture, she experienced pain with forward flexion of her hip to 30°. Imaging revealed defects from the previous hardware. The left subtrochanteric femoral stress fracture was fixed with a blade plate, which was removed two years later due to symptomatic hardware.

**Figure 1:**
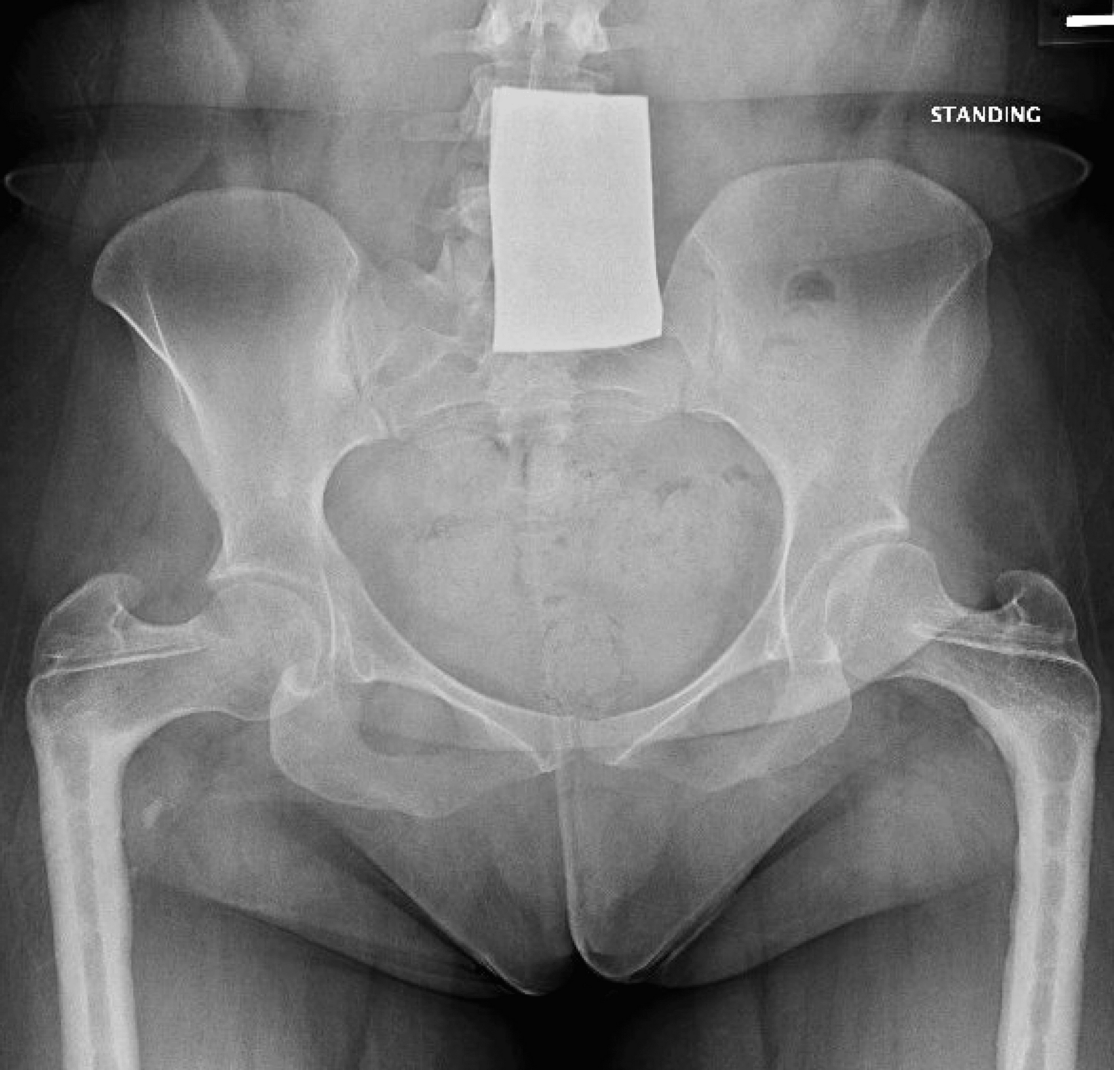
Pre-fracture X-ray showing the neck angle and deformities from the removed hardware

**Figure 2:**
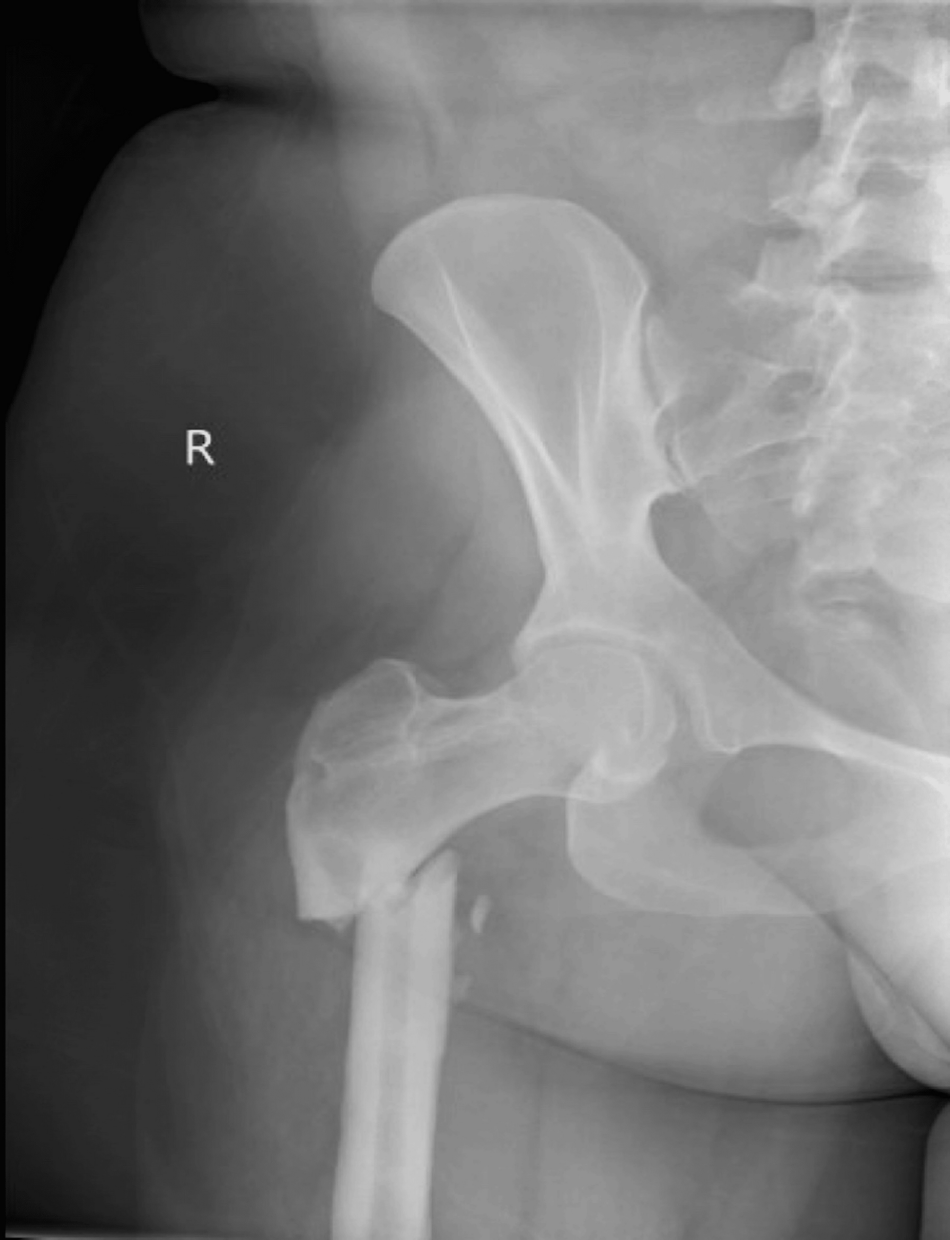
Subtrochanteric right femur fracture

The patient subsequently sustained a low-energy right subtrochanteric fracture. Imaging from four years prior to the fracture, the anterior-to-posterior (AP) pelvis view showed both femoral heads reduced with healed 95° varus osteotomies (Figure 1). Imaging at the time of the fracture revealed an acute, displaced fracture of the proximal femoral diaphysis (Figure 2). The fracture propagated through a hole in the femur from a previously removed screw. She had a 98° neck-shaft angle and 6-mm medullary canal. Given these factors, the decision was made to fix this fracture with a humeral nail. The treating surgeon determined that repeat plate fixation would likely fail due to the extreme femoral neck-shaft angle, which would create significant angular forces on the proximal femur, poorly controlled by a surface-based implant. A medullary device, with its orientation closely aligned to that of the femur, would more likely promote healing before fracture failure. However, because the neck-shaft angle was less than 100°, a traditional cephalomedullary nail would not match the patient’s anatomy. Therefore, a humeral nail, with a 90° neck-shaft angle, was chosen as it would more closely match the patient’s anatomy. 

**Figure 3:**
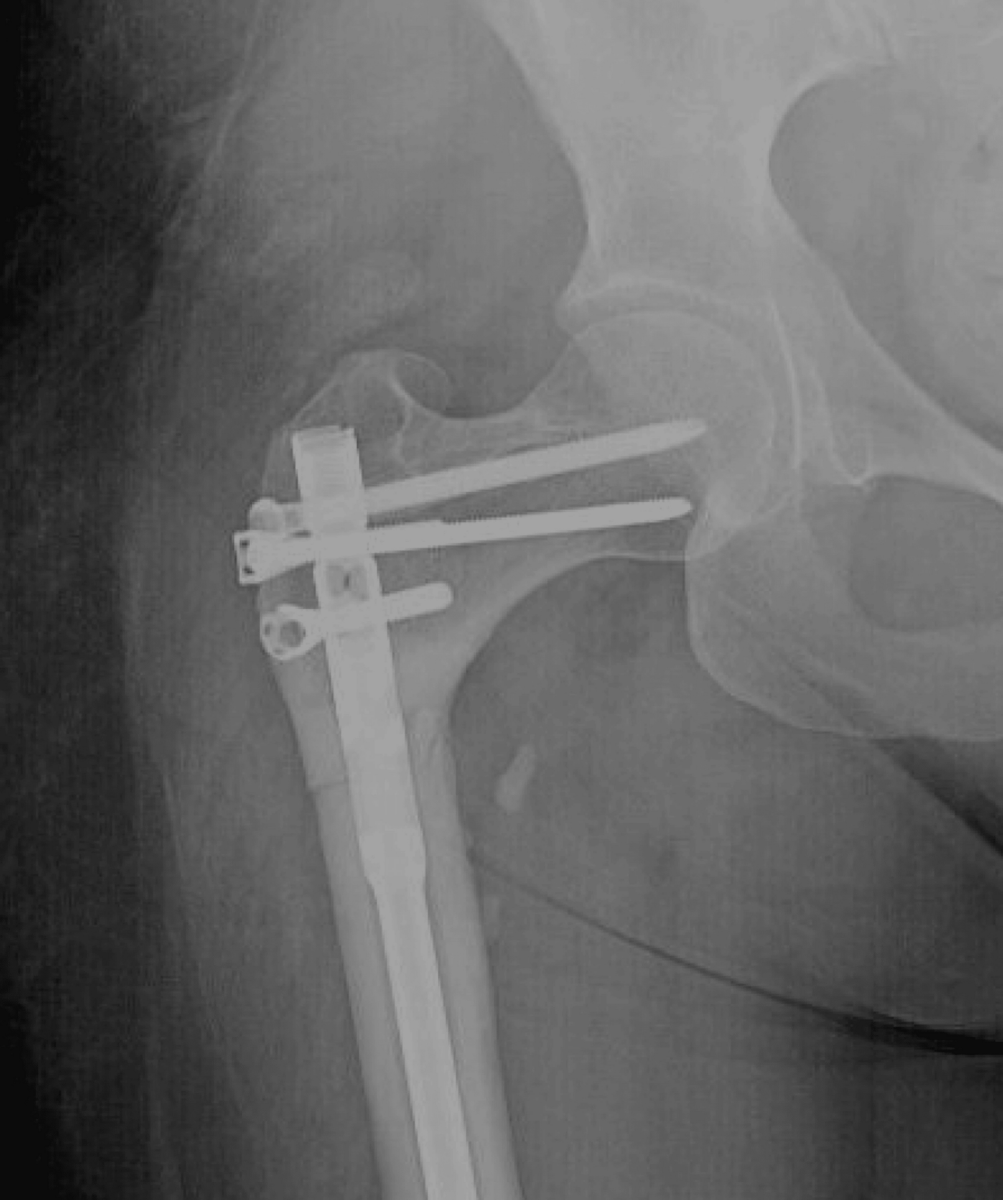
Post-op imaging of the right hip with a humeral nail

**Figure 4:**
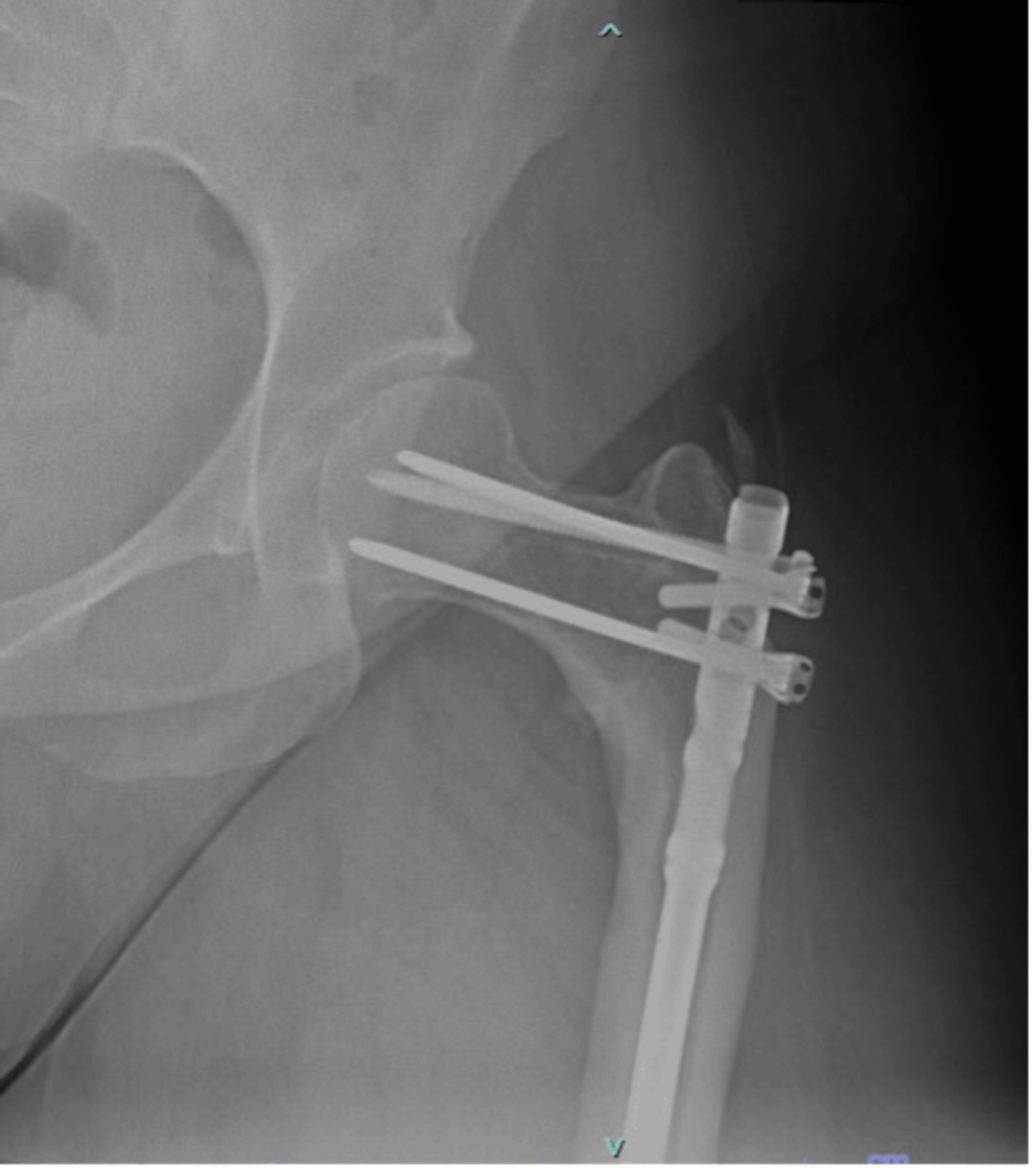
Post-op imaging of the left hip with a humeral nail

**Figure 5:**
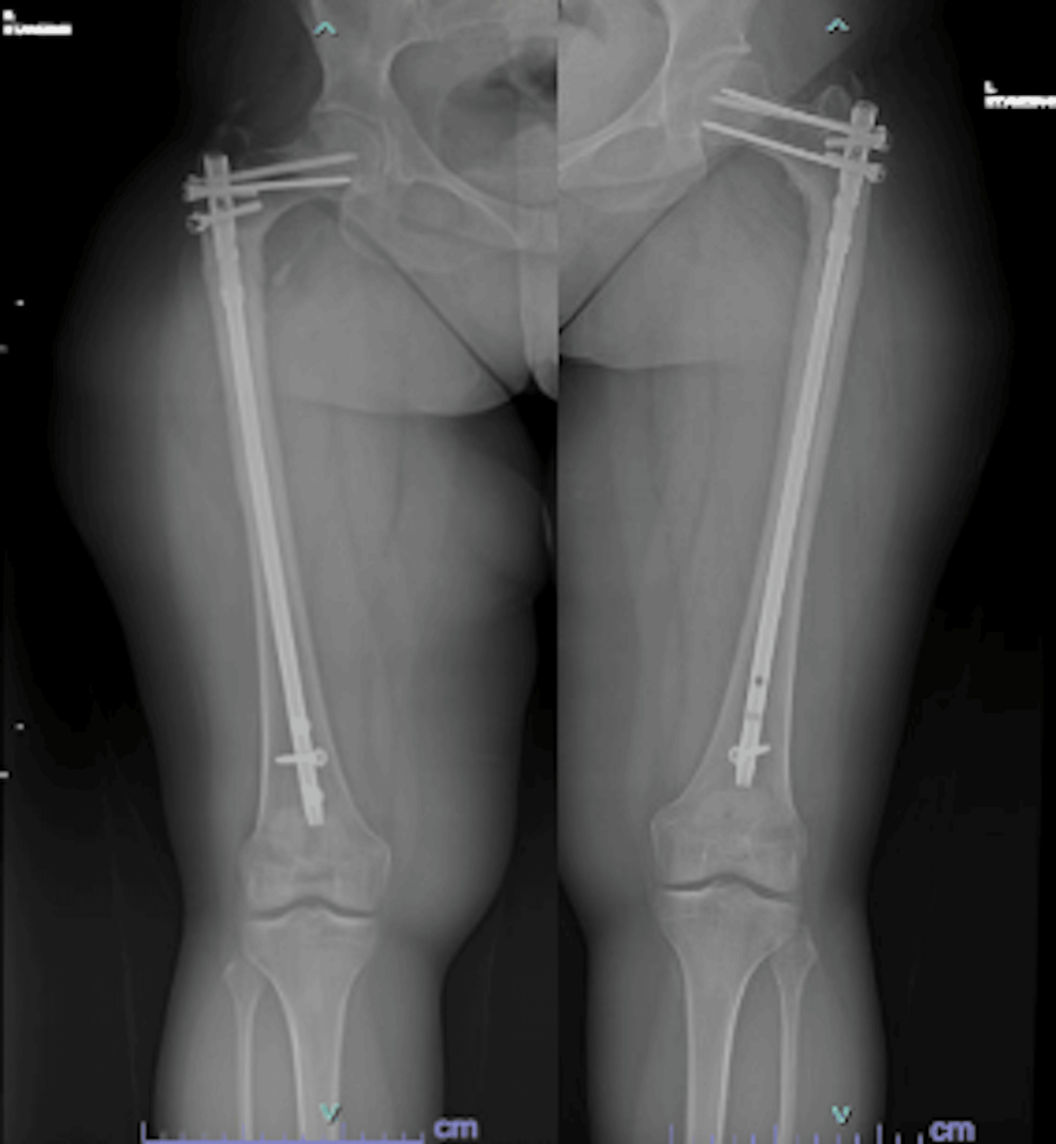
Most recent imaging showing radiographically healed fracture and intact hardware

The patient underwent open reduction and internal fixation of the right subtrochanteric femur fracture using an intramedullary Synthes MultiLoc Humeral Nail (7 mm x 300 mm; Synthes USA Products, West Chester, USA), with multiple 4.5-mm MultiLoc screws, a 3.5-mm Synthes locking screw, and a 4.5-mm Smith and Nephew interlocking bolt (Smith & Nephew, London, UK). The subtrochanteric fracture was reduced through an open incision under direct visualization with bone clamps. The medullary canal was reamed, starting with a 6-mm end-cutting humeral reamer and increased sequentially in 0.5-mm increments up to 10.5 mm. Due to the nail’s anatomy distally, a left-sided humeral nail was used for the right femur to allow for interlocking bolt placement. The length of the nail was selected to protect the entire femur, specifically in the supracondylar region, to prevent future fractures from the patient’s prior femoral instrumentation. The straight nail was contoured using a large tabletop bender to create an anterior bow, matching the patient’s estimated femoral radius of curvature. Three 4.5-mm MultiLoc interlocking bolts were placed into the femoral neck and head. Proximally, an off-axis 3.5-mm locking bolt and a 4.5-mm Smith and Nephew interlocking bolt were inserted. The nail was locked distally with three interlocking bolts using the perfect circles technique. The patient had an uneventful postoperative course and was discharged home. Postoperative imaging demonstrated anatomic alignment and solid fixation (Figure 3). At six months postoperatively, CT imaging showed approximately 50% cortical bridging medially. The patient reported persistent achiness and pain in the right hip, raising concerns of delayed union or nonunion. After a discussion with the patient, the decision was made to proceed with prophylactic stabilization of the left proximal femur using a 7 mm x 300 mm Synthes MultiLoc humeral nail, along with a 4.5-mm Smith and Nephew titanium interlocking bolt, at the same time as the right femur nonunion repair. A right-sided nail was used for the left femur, and the nail was contoured to match the patient’s radius of curvature. The medullary canal was reamed to 10 mm with a reamer irrigator aspirator to harvest bone graft for the contralateral nonunion repair. A single interlocking bolt was placed proximally through the nail, and two MultiLoc bolts were placed proximally, each secured with 3.5-mm stainless steel locking screws (Figure 4). Distally, a single AP 4-mm interlocking bolt was inserted. For the right proximal femur nonunion repair, the nonunion site was thoroughly debrided and packed with the bone graft harvested from the contralateral side reaming, mixed with 1 g of vancomycin powder. The patient was discharged home, and the postoperative course was uneventful. Over two years later, the patient is clinically and radiographically healed from her subtrochanteric femur fracture (Figure 5).

## Discussion

The use of humeral nails for subtrochanteric femur fractures is rarely reported in the literature. Standard femoral nails may present challenges in treating patients with abnormal anatomy, such as altered femoral neck angles or narrow medullary canal diameters. Blade plate implants serve as an alternative for stabilizing subtrochanteric fractures in such patients, but these come with multiple downsides, such as extended time to weight bearing, increased soft tissue disruption, and challenging surgical technique [1]. 

Published literature describing the off-label use of humeral nails is limited. Peters et al. [7] describe the case of a 17-year-old paraplegic patient with a subtrochanteric femur fracture where an intramedullary femoral nail was not possible due to anatomical conditions. In this case, a humeral nail was chosen to avoid operation-related soft tissue damage, achieve positional stability, and prevent internal pressure ulcers. 

Sa-Ngasoongsong et al. [8] describe a series of patients with osteogenesis imperfecta who successfully underwent femoral fixation with a humeral nail. Although these cases resulted in favorable outcomes, the authors noted several limitations to using a humeral nail for femoral reconstruction. These included the need for extra-length distal locking screws and the 90-100° cephalomedullary angle required for proximal locking screws. However, in our case, this angle was favorable due to the patient’s previous femoral varus osteotomies. 

Gordon et al. [9] conducted a retrospective review of using humeral intramedullary nails to treat pediatric femoral shaft fractures. Similarly, Park et al. [6] performed a retrospective review of children and adolescents who underwent rigid intramedullary nailing using a humeral nail for femoral shaft fractures in regions where pediatric rigid nails were not available. Both studies found success with humeral nails when pediatric femoral nails were not an option. 

## Conclusions

There are limited options for addressing subtrochanteric femur fractures in patients with anatomy that precludes the use of standard femoral nails. This case presents one potential solution. Our patient, with a history of varus-producing proximal femoral osteotomies, sustained a subtrochanteric femur fracture. Given her irregular neck-shaft angle and narrow intramedullary canal, a humeral nail was chosen for fracture fixation. She subsequently underwent prophylactic stabilization of the contralateral femur with a humeral nail. This case highlights the potential use of humeral nails in femoral fractures, particularly in patients with challenging anatomical considerations, and may serve as an example for future cases where this approach is warranted.

## References

[1] Lundy DW (2007). Subtrochanteric femoral fractures. J Am Acad Orthop Surg.

[2] Gänsslen A, Gösling T, Hildebrand F, Pape HC, Oestern HJ (2014). Femoral shaft fractures in adults: treatment options and controversies. Acta Chir Orthop Traumatol Cech.

[3] Wang YQ, Hu YC, Xu ZM, Zhao YW, Wu JM (2009). An intramedullary nail with multifunctional interlocking for all types of fracture in both femurs. Orthop Surg.

[4] Pires RE, Yoon RS, Liporace FA (2020). Expanding the horizons of clinical applications of proximal humerus locking plates in the lower extremities: a technical note. Chin J Traumatol.

[5] He S, Yan B, Zhu J, Huang X, Zhao J (2018). High failure rate of proximal femoral locking plates in fixation of trochanteric fractures. J Orthop Surg Res.

[6] Park H, Kim HW (2012). Treatment of femoral shaft fracture with an interlocking humeral nail in older children and adolescents. Yonsei Med J.

[7] Peters J, Köhler HC, Gutcke A, Schulze C (2022). Fixing a subtrochanteric femoral fracture with a humerus nail. Ortop Traumatol Rehabil.

[8] Sa-Ngasoongsong P, Saisongcroh T, Angsanuntsukh C, Woratanarat P, Mulpruek P (2017). Using humeral nail for surgical reconstruction of femur in adolescents with osteogenesis imperfecta. World J Orthop.

[9] Gordon JE, Khanna N, Luhmann SJ, Dobbs MB, Ortman MR, Schoenecker PL (2004). Intramedullary nailing of femoral fractures in children through the lateral aspect of the greater trochanter using a modified rigid humeral intramedullary nail: preliminary results of a new technique in 15 children. J Orthop Trauma.

